# Supplementary material for: High acceptability of newborn screening for sickle cell disease among post-natal mothers in Western Kenya
Source: PLoS One. 2025 Jul 24;20(7):e0305156. doi: 10.1371/journal.pone.0305156 (PMC12288990; doi:10.1371/journal.pone.0305156)
Supplement: Attachment — Submitted filename: RESPONSE TO REVIEWERS.docx [file pone.0305156.s003.docx]

P.O Box 210-40601

To the academic editor,

PLOS ONE journal,

18/10/2024

Dear Sir/Madam,

RE: RESPONSE TO THE REVIEWERS COMMENTS

Thank you for the thorough review of this manuscripts. Please find the responses to the comments raised.

Additionally provided are the clean and tracked copies of the manuscript

I will gladly appreciate review of the same and or its publication.

The authors have no conflict of interest.

Thank you.

Yours faithfully,

John Orimbo,

Corresponding author.

When submitting your revision, we need you to address these additional requirements.

1. Please ensure that your manuscript meets PLOS ONE's style requirements, including those for file naming. The PLOS ONE style templates can be found at 
<https://journals.plos.org/plosone/s/file?id=wjVg/PLOSOne_formatting_sample_main_body.pdf> and 
<https://journals.plos.org/plosone/s/file?id=ba62/PLOSOne_formatting_sample_title_authors_affiliations.pdf>

**Response: The manuscript has been updated to meet the PLOS ONE’s style requirements.**

2. We note that the grant information you provided in the ‘Funding Information’ and ‘Financial Disclosure’ sections do not match.

**Response: This has been harmonized**

When you resubmit, please ensure that you provide the correct grant numbers for the awards you received for your study in the ‘Funding Information’ section.

3. Thank you for stating the following in your Competing Interests section: None

Please complete your Competing Interests on the online submission form to state any Competing Interests. If you have no competing interests, please state ""The authors have declared that no competing interests exist."", as detailed online in our guide for authors at <http://journals.plos.org/plosone/s/submit-now>

 This information should be included in your cover letter; we will change the online submission form on your behalf.

**Response: Thank you, this has been included**

4. Please provide a complete Data Availability Statement in the submission form, ensuring you include all necessary access information or a reason for why you are unable to make your data freely accessible. If your research concerns only data provided within your submission, please write "All data are in the manuscript and/or supporting information files" as your Data Availability Statement.

**Response: Data will be availed via a link**

5. Your ethics statement should only appear in the Methods section of your manuscript. If your ethics statement is written in any section besides the Methods, please move it to the Methods section and delete it from any other section. Please ensure that your ethics statement is included in your manuscript, as the ethics statement entered into the online submission form will not be published alongside your manuscript.

**Response: This has been moved to the methods section**

6. Please review your reference list to ensure that it is complete and correct. If you have cited papers that have been retracted, please include the rationale for doing so in the manuscript text, or remove these references and replace them with relevant current references. Any changes to the reference list should be mentioned in the rebuttal letter that accompanies your revised manuscript. If you need to cite a retracted article, indicate the article’s retracted status in the References list and also include a citation and full reference for the retraction notice.

**Response: This has been reviewed and also cited references left**

[Note: HTML markup is below. Please do not edit.]

Reviewers' comments:

Reviewer's Responses to Questions

**Comments to the Author**

1. Is the manuscript technically sound, and do the data support the conclusions?

The manuscript must describe a technically sound piece of scientific research with data that supports the conclusions. Experiments must have been conducted rigorously, with appropriate controls, replication, and sample sizes. The conclusions must be drawn appropriately based on the data presented.

Reviewer #1: Yes

Reviewer #2: Yes

2. Has the statistical analysis been performed appropriately and rigorously?

Reviewer #1: Yes

Reviewer #2: Yes

3. Have the authors made all data underlying the findings in their manuscript fully available?

The [PLOS Data policy](http://c05y1x9s.r.us-east-2.awstrack.me/L0/http:%2F%2Fwww.plosone.org%2Fstatic%2Fpolicies.action%23sharing/1/010f01914c7eba97-1cd98774-a407-464f-9bbf-5db558545c4d-000000/4TFNKaoDYBlgvh2M4A4vxlM8WeM=171) requires authors to make all data underlying the findings described in their manuscript fully available without restriction, with rare exception (please refer to the Data Availability Statement in the manuscript PDF file). The data should be provided as part of the manuscript or its supporting information, or deposited to a public repository. For example, in addition to summary statistics, the data points behind means, medians and variance measures should be available. If there are restrictions on publicly sharing data—e.g. participant privacy or use of data from a third party—those must be specified.

Reviewer #1: No

Reviewer #2: No

4. Is the manuscript presented in an intelligible fashion and written in standard English?

PLOS ONE does not copyedit accepted manuscripts, so the language in submitted articles must be clear, correct, and unambiguous. Any typographical or grammatical errors should be corrected at revision, so please note any specific errors here.

Reviewer #1: Yes

Reviewer #2: Yes

5. Review Comments to the Author

Please use the space provided to explain your answers to the questions above. You may also include additional comments for the author, including concerns about dual publication, research ethics, or publication ethics. (Please upload your review as an attachment if it exceeds 20,000 characters)

Reviewer #1: well written manuscript. Some comments: -
1. Introduction- Could you highlight the national government newborn screening policy on SCD? This is referenced in the methods and discussion section.

**Response: This has been added to the abstract to read, “The Kenyan ministry of health, in 2020, published a policy on newborn screening for sickle cell disease from levels 2 to 6 healthcare facilities.”**

2. Its likely the data are clustered around the study facility. Its important to provide the geographical location of the participants - the data shown in the discussion under limitations 'with each subcounty contributing at least 5% of the
369 total sample has not been provided.

**Response: This has now been provided in the table 1 of the results section under the sociodemographic characteristics of the participants**

3. Consider dropping this phrase 'at Homabay County Teaching and Referral Hospital, western Kenya' to improve readability

**Response: This has been dropped in all other parts except the abstract where it is retained to explain the study setting**

4. Consider dropping figure 1 as it does not add much value

**Response: Figure one has been dropped**

5. What informed the questions on acceptability? They seem narrow focusing on beliefs. There might be other key aspects of acceptability that these narrowly focused questions would not evaluate. its also not clear how responses to these questions could inform improved acceptability in other areas.

**Response: The questions were largely informed by the review of literature on the previous studies on the factors that have influenced user acceptability especially at user level and guided by the theoretical framework of acceptability. Other factors like access and provider attitude which affect majorly concurrent acceptability were not explored in this study since newborn screening is not yet implemented in the county.**

6. Screening Barriers: Could you comment on why, despite high acceptance and knowledge of SCD screening, the majority of the mothers reported not having been screened? What could be the barriers to screening that would need specific recommendations?

**Response: This has been added to the discussion section on paragraph 3 to read, “Further, despite the high acceptance and good knowledge on screening for sickle cell disease among the postnatal mothers who were interviewed, only few of the mothers had been screened for sickle cell disease. This was mainly due to the fact that routine screening for sickle cell disease has not been adopted in the county, and including among newborns, and thus only those with symptoms of sickle cell disease are tested or screened for the disease.”**

7. was the effect of clustering in time and space explored in this study

**Response: No, this was not explored**

8 The discussion needs to be revised to be succinct and clear-- a maximum of two pages

**Response: This has been revised**

9. Read the paper to correct grammar and appropriate punctuations

**Response: Grammar and punctuations have been corrected across the paper.**

Reviewer #2: Summary
The authors assessed acceptability of newborn screening for Sickle Cell disease among 399 post-natal mothers at Homa Bay county hospital in western Kenya. They focused on assessing level of acceptability as a step in the pathway towards programmatic scale up of newborn screening.
Mothers who chose to participate were consented and interviewed using a structured questionnaire and their newborns were screened using Sickle SCAN point-of-care test.
Acceptability of screening was high at 94%. Being a student was associated with a higher likelihood of accepting screening. The authors recommended that Homabay county and the Kenyan ministry of health should implement routine newborn screening for sickle cell disease in all level 2-6 hospitals.

General Comment
The manuscript is clearly written and addresses an important topic.
It could however benefit from some edits. Authors should pay particular attention to data analysis and interpretation of results on factors associated with acceptability screening.

Abstract
1. Methods (Line 21-27): It is not clear whether potential participants were empowered with information on the benefits of screening newborns for SCD before being invited to make an informed choice to enroll in the study.

**Response: All the potential participants were taken through written informed consent prior to participation. The informed consent had both the risks and benefits of participation in the study, including of doing screening for sickle cell disease on the newborn, and this were adequately explained to the participant prior. Only participants who were willing after being taken through the informed consent signed the consent and were subsequently enrolled. This thus met the threshold of adequate informed to allow them make an informed decision. This has been added to the abstract to read, “After obtaining informed consent, a semi-structured questionnaire was used for data collection.”**

2. Conclusion (Line 35-36): The second sentence is directive and not fully supported by the results. Why all level 2-6 hospitals? Consider editing this sentence to improve its clarity.

**Response: This has been revised to read, “The Homabay county ministry of health should implement routine newborn screening for sickle cell disease in all health care facilities conducting deliveries of newborns”. This is since the study was conducted among post delivery mothers, it can be extrapolated for all healthcare facilities conducting deliveries across the county.**

Background
It is stated that the prevalence of sickle cell disease and sickle cell trait in western Kenya is 4.5% and 18%, respectively. Please specify age band for reported estimates.

**Response: This statement has been revised to read, “The prevalence of sickle cell disease around western Kenya at birth is about 4.5% for sickle cell disease, and 18% for sickle cell trait”**


Methods
It is stated that Minor mothers also were provided with a signed assent… edit this to improve clarity. Specify what was done. Assented and signed an assent form or assented verbally and someone else signed a consent form on their behalf?

**Response: This has been edited to read, “Written informed consent was obtained from potential participants who were 18years and above. A written informed assent was obtained from minor mothers (those less than 18 years and lacking legal capacity to consent on their own) in addition to a written consent from their parents or guardians.”**

There are no details of how mothers with no prior knowledge on sickle disease were empowered with information to make informed choice about participating in the study is missing. Consider editing to include the missing information.

**Response: This information was provided to the mothers through the informed consent and assent and has now been added to read,** **“Written informed consent was obtained from potential participants who were 18years and above. A written informed assent was obtained from minor mothers (those less than 18 years and lacking legal capacity to consent on their own) in addition to a written consent from their parents or guardians. The informed consents and assents were adequate details on sickle cell disease and their participation in the study, and also on risk and benefits of participation. This was made so in order to provide the potential participants with adequate prior information for making an informed decision before signing the informed consent or assent.”**

Line 127: To is misplaced within the sentence: Consider deleting.

**Response: This has been deleted**

Results
Line 191: Replace “belived” with believed.

**Response: The spelling has been corrected to believed.**

The confidence intervals for some estimates are uncomfortably wide thus undermining the utility of some results. Please review. E.g. line 32-33: being a student (aOR= 25.02; 95% CI=1.29-484.51; p= 0.033).

**Response: This has been reviewed**
Table 5:
1. Review choice of reference group for population categories in table 5.

**Response: This has been reviewed**

2. The authors have only used 4 variables to check associations based on chi square tabulations instead of using all the variables in the unadjusted model to determine those to be used in adjusted model.

**Response: Only the variables that had a statistically significant association with the acceptability of newborn screening for sickle cell disease were moved to unadjusted model to determine the strength of association thus only 4 variables moved.**

3. Overall, the very wide confidence intervals may undermine the practical applications of results touching on the predictors acceptability.

**Response: This has been reviewed upon review of the choice of reference group**

6. PLOS authors have the option to publish the peer review history of their article ([what does this mean?](https://c05y1x9s.r.us-east-2.awstrack.me/L0/https:%2F%2Fjournals.plos.org%2Fplosone%2Fs%2Feditorial-and-peer-review-process%23loc-peer-review-history/1/010f01914c7eba97-1cd98774-a407-464f-9bbf-5db558545c4d-000000/Xi5eFV-PVbfY58k6Vrj5Acvr2q8=171)). If published, this will include your full peer review and any attached files.


If you choose “no”, your identity will remain anonymous but your review may still be made public.


**Do you want your identity to be public for this peer review?** For information about this choice, including consent withdrawal, please see our [Privacy Policy](https://c05y1x9s.r.us-east-2.awstrack.me/L0/https:%2F%2Fwww.plos.org%2Fprivacy-policy/1/010f01914c7eba97-1cd98774-a407-464f-9bbf-5db558545c4d-000000/J-9lH_eyRpxGOIKwDL27MrBcAns=171).

Reviewer #1: **Yes:** Peninah Munyua

Reviewer #2: No
