# Supplementary material for: High acceptability of newborn screening for sickle cell disease among post-natal mothers in Western Kenya
Source: PLoS One. 2025 Jul 24;20(7):e0305156. doi: 10.1371/journal.pone.0305156 (PMC12288990; doi:10.1371/journal.pone.0305156)
Supplement: Attachment — Submitted filename: Response to Reviewers John.docx [file pone.0305156.s004.docx]

 Introduction - Are there no studies in literature on acceptability of newborn screening at all?  You may please consider and review articles such as the article by “Nnachi OC et. Acceptability of Newborn Screening for Sickle Cell Disease among Post-Partum Mothers in Abakaliki, South East Nigeria. West Afr J Med. 2023 Mar 31;40(3):298-304. PMID: 37017939.” And other related literature.

**Response: Thank you for the input. However, the introduction reads. “There is however, paucity of data on the acceptability of newborn screening in the county” This implies there has not been any study previous assessing acceptability in Homa bay county. We however acknowledge that several studies have been done across sub-Saharan Africa on acceptability of newborn screening for sickle cell disease.**

    Methods – Line 117 - Did you obtain assent from children? Assent is not the same as consent. Your manuscript implies they are similar. Please clarify what you did. Assent is usually obtained from minors, and consent from adults.

**Response: Consent was obtained from adult mothers. For minor mothers, they signed an assent and their parent or guardian signed the consent. This has now been clarified.**
    Methods – Line 131 – 133. There’s need to describe the government policy in a section in the initially under methods.

**Response: Thank you. The policy has been explained under the introduction section.**
    Methods – Linie 181 - Assent is not the same as consent.

**Response: Thank you, this has been clarified.**

    Discussion – Line 314 – 425.

Please revise the discussion section. Kindly write this section in a systematic, focused manner. i. Start with a Summary of Key Findings (May be 2-3 key findings). ii. Interpret the Findings. iii. Compare with Existing Literature. Iv. Explain the Strengths and Limitations. v. Discuss Implications for Practice or Policy. vi. Offer Recommendations or Future Research Directions. vii.Write a clear conclusion.

While most elements of the discussion are present in the Discussion Section in its present form, the discussion section will benefit from a re-organisation.

**Response: This has been revised appropriately as guided.**

In addition, the authors are also advised to address the peer reviewer's comments below:

"Summary
The authors assessed acceptability of newborn screening for Sickle Cell disease among 399 post-natal mothers at Homa Bay county hospital in western Kenya. They focused on assessing level of acceptability as a step in the pathway towards programmatic scale up of newborn screening.
Mothers who chose to participate were consented and interviewed using a structured questionnaire and their newborns were screened using Sickle SCAN point-of-care test.
Acceptability of screening was high at 94%. Being a student was associated with a higher likelihood of accepting screening. The authors recommended that Homabay county and the Kenyan ministry of health should implement routine newborn screening for sickle cell disease in all level 2-6 hospitals.

General Comment
The manuscript is clearly written and addresses an important topic.
It could however benefit from some edits. Authors should pay particular attention to data analysis and interpretation of results on factors associated with acceptability screening.

Abstract
1. Methods (Line 21-27): It is not clear whether potential participants were empowered with information on the benefits of screening newborns for SCD before being invited to make an informed choice to enroll in the study.

**Response: The potential participants were provided with the information through the informed consent documents which they were taken through by a research assistant in a language of their choice before making informed decision to enrol. The information included benefits of screening the newborn, result interpretation, linkages to care and possible risks of participation in the study.**
2. Conclusion (Line 35-36): The second sentence is directive and not fully supported by the results. Why all level 2-6 hospitals? Consider editing this sentence to improve its clarity.

**Response: This has been edited to read, “The acceptability of newborn screening for sickle cell disease is high in the county.** **The Homabay county ministry of health should implement routine newborn screening for sickle cell disease in all healthcare facilities conducting deliveries of newborns.”**

Background
It is stated that the prevalence of sickle cell disease and sickle cell trait in western Kenya is 4.5% and 18%, respectively. Please specify age band for reported estimates.

**Response: This has been clarified to read,** **“The prevalence of sickle cell disease around western Kenya at birth is about 4.5% for sickle cell disease, and 18% for sickle cell trait.”**

Methods
It is stated that Minor mothers also were provided with a signed assent… edit this to improve clarity. Specify what was done. Assented and signed an assent form or assented verbally and someone else signed a consent form on their behalf?

There are no details of how mothers with no prior knowledge on sickle disease were empowered with information to make informed choice about participating in the study is missing. Consider editing to include the missing information.

**Response: This has been added in the methods section under the sub topic enrolment and data collection, lines 184-193**

Line 127: To is misplaced within the sentence: Consider deleting.

**Response: This was deleted.**

Results
Line 191: Replace “belived” with believed.

**Response: This was updated accordingly**
The confidence intervals for some estimates are uncomfortably wide thus undermining the utility of some results. Please review. E.g. line 32-33: being a student (aOR= 25.02; 95% CI=1.29-484.51; p= 0.033).

**Response: This was reviewed to “(aOR= 0.04; 95% CI=0.00-0.78*;* *p*= 0.033)” after re-analysis**

Table 5:
1. Review choice of reference group **for population categories in table 5.**

**Reviewed: This has been reviewed.**
2. The authors have only used 4 variables to check associations based on chi square tabulations instead of using all the variables in the unadjusted model to determine those to be used in adjusted model.

**Response: All the variables were subject ton test of association using either chi-square or fisher’s exact, where criteria for chi-square was not met. Only the variables that had an association with acceptability of newborn screening for sickle cell disease were subjected with univariate logistic regression (unadjusted model) in order to determine the strength of association thus not all variables were used for the unadjusted model. This was as per the approved protocol statistical analysis plan.**
3. Overall, the very wide confidence intervals may undermine the practical applications of results touching on the predictors acceptability."

**Response: upon re-analysis, the confidence intervals are now much better. However, this can be attributed to slightly smaller sample size even though our sample size was larger than the calculated sample size.**
