# Supplementary material for: High acceptability of newborn screening for sickle cell disease among post-natal mothers in Western Kenya
Source: PLoS One. 2025 Jul 24;20(7):e0305156. doi: 10.1371/journal.pone.0305156 (PMC12288990; doi:10.1371/journal.pone.0305156)
Supplement: Attachment — Submitted filename: Response_to_reviewers_auresp_3.docx [file pone.0305156.s005.docx]

Rebuttal letter

**Please revise the title of the manuscript as follows and resubmit:**

**"High Acceptability of Newborn Screening for Sickle Cell Disease Among Post-Natal Mothers in Western Kenya"**

**Response: This has been updated as requested**
